# Microvascular decompression for the treatment of neurogenic hypertension with trigeminal neuralgia

**DOI:** 10.1186/s12883-019-1569-y

**Published:** 2019-12-27

**Authors:** Wenchao Lu, Hui Wang, Zhongnan Yan, Yuangang Wang, Hongmin Che

**Affiliations:** 1 Department of Neurosurgery, the Xi’an Gaoxin Hospital, No.16 Tuanjie South Road, Xi’an, 710061 Shaanxi Province China; 2 Department of Neurosurgery, the Xi’an Daxing Hospital, Xi’an, Shaanxi Province China

**Keywords:** Neurogenic hypertension, microvascular decompression, Trigeminal neuralgia, Rostral ventrolateral medulla

## Abstract

**Background:**

To evaluate the efficacy of microvascular decompression (MVD) in reducing hypertension (HTN) in hypertensive patients with trigeminal neuralgia (TN).

**Methods:**

The clinical data of 58 cases of neurogenic HTN with TN treated in our hospital were retrospectively reviewed. Preoperative MR revealed abnormal blood pressure in the left rostral ventrolateral medulla (RVLM) and the posterior cranial nerve root entry zone (REZ). The patients were divided into control group: only trigeminal nerve was treated with MVD; experimental group: trigeminal nerve, RVLM and REZ were treated with MVD at the same time. The patients were followed up for 6 months to 1 year to observe the changes of blood pressure.

**Results:**

There was no significant difference in gender, age, course of TN, course of HTN, grade of HTN and preoperative blood pressure between the two groups. After operation, the effective rate of HTN improvement with MVD was 32.1% in the control group. There was no significant difference in the preoperative and post operative blood pressure. (P△_SBP_ = 0.131; P△_BDP_ = 0.078). In the experimental group, the effective rate was 83.3%. The postoperative blood pressure was significantly lower than preoperative values. (P△_SBP_ < 0.001; P△_DBP_ < 0.001).

**Conclusions:**

MVD is an effective treatment for neurogenic HTN. However, the criteria for selecting hypertensive patients who need MVD to control their HTN still needs to be further determined. Possible indications may include: left trigeminal neuralgia, neurogenic HTN; abnormal blood pressure compression in the left RVLM and REZ areas on MR; and blood pressure in these patients can not be effectively controlled by drugs.

## Background

With the improvement of people’s living standards and the aging of our society, HTN is one of the diseases that seriously threaten human health, its incidence is increasing year by year. If there is no effective treatment, it often leads to serious complications and even death [1]. Most patients with HTN can get ideal control of blood pressure through systematic and regular medication. However, some patients still have difficulty to effectively control blood pressure after combined antihypertensive drugs. Jannetta et al. [2] suggested that the cause of HTN may be due to the left rostral ventrolateral medulla (RVLM) and the REZ. Due to pulsatile compression, if the vascular compression is relieved, HTN can be effectively relieved and treated. At present, microvascular decompression (MVD) has become a special treatment for TN by separating the responsible vessels from the nerves and relieving the compression of the blood vessels [3]. However, it is difficult to select patients for MVD only because of HTN. We retrospectively analyzed the clinical outcomes of 58 neurogenic HTN with TN undergoing MVD. Among them, 30 patients underwent MVD in the left RVLM and REZ regions simultaneously. Satisfactory results were achieved after operation.

## Methods

### Inclusion and exclusion criteria

1. Inclusion criteria: ①Primary left TN with typical clinical presentation, without any specific etiology determined. ②According to criteria for diagnosis of HTN, a baseline systolic pressure greater than 140 mmHg and a baseline diastolic pressure greater than 90 mmHg [4]. ③The combined use of antihypertensive drugs (calcium antagonists, diuretics, angiotensin converting enzyme inhibitors and beta-receptor antagonists) has poor therapeutic effect. ④Preoperative MR confirms the presence of vascular compression in the left RVLM and REZ regions.
2. Exclusion criteria: ① Secondary TN; ② Secondary HTN, including renal HTN, endocrine HTN and drug-induced HTN; ③Combined with heart failure or arrhythmia, antihypertensive drugs cannot be stopped; ④Alcoholism, drug abuse, mental disorders, irregular life; ⑤Oral antihypertensive drugs are irregular; ⑥Other surgical contraindications for MVD.

### General situation

From January 2009 to October 2018, 58 patients with left TN with HTN were included. After admission, blood pressure was monitored for at least three consecutive days. Preoperative oral or intravenous medication was used to control blood pressure at an acceptable level during surgery. (systolic blood pressure < 150 mmHg, diastolic blood pressure < 95 mmHg). Among them, 30 patients (17 males and 13 females, aged 60.6 ± 9.4 years) received microvascular decompression of the left trigeminal nerve and RVLM and REZ area as experimental group with the consent of the patients themselves and their families, while microvascular decompression of the left trigeminal nerve was performed only in 28 patients (18 males and 10 females, aged 61.2 ± 9.6 years) as the control group.

### Imaging examination

All patients underwent preoperative MRI scanning. The relationship between blood vessels and trigeminal nerve, RVLM and REZ was evaluated by three-dimensional time-of-flight magnetic resonance angiography (3D-TOFMRA). If there was a possibility of contact or compression, it was positive for MRI. Two radiologists would evaluate the results individually. When the evaluation results were inconsistent, it would be decided after discussion.

### Surgical methods

All patients were treated by left suboccipital retrosigmoid approach. Routine craniotomy was performed to expose the posterior edge of sigmoid sinus and the inferior edge of transverse sinus. The open mastoid air chamber was sealed with bone wax to prevent cerebrospinal fluid fistula. The dura mater was cut off and sharp separation was performed under the operating microscope. The occipital cistern, cerebellomedullary cistern and pontine cistern were opened. In the control group, only trigeminal nerve decompression was performed to free the responsible artery, and Teflon cotton was inserted to isolate it from the initial segment of the brain nerve root. In the experimental group, the trigeminal nerve was decompressed, the responsible artery was free, and the appropriate size of Teflon cotton was inserted to isolate the trigeminal nerve from the initial segment of the brain nerve root. In the same way, RVLM and REZ areas were explored. According to the relationship between vessels and RVLM and REZ areas, the responsible arteries were isolated and separated from the initial segment of the brain nerve root by inserting Teflon cotton of appropriate size. There was no obvious bleeding in the operative field and the dural incision was tightly sutured and the muscles, cap aponeurosis and scalp were sutured layer by layer. All operations were performed by the same surgical team.

### Blood pressure monitoring

(1) After admission, the blood pressure under the condition of no medication was monitored in the morning for at least three consecutive days. (after completing blood pressure measurement, routine medication was continued). The mean blood pressure was taken as preoperative blood pressure. (2) Blood pressure in the morning without medication as monitored for 3 consecutive days in each review month (1 month, 3 months, 6 months and 12 months) after surgery respectively. The mean blood pressure was taken as the blood pressure of the review month. (3) After operation, blood pressure was measured three times in different days without change of taking antihypertensive drugs. If systolic blood pressure (SBP) < 140 mmHg or diastolic blood pressure (DBP) < 90 mmHg, Antihypertensive drugs start to reduce or even stop drugs.

### Efficacy evaluation

According to the extent of blood pressure decline and the degree of relief of symptoms, the therapeutic effect was judged as follows: ①. Cure: after the patient completely stopped using antihypertensive drugs, systolic blood pressure < 140 mmHg, and diastolic blood pressure < 90 mmHg; ②. marked effect: systolic and diastolic blood pressure, one of which decreased to normal level or systolic blood pressure < 30 mmHg and diastolic blood pressure decreased < 10 mmHg; ③. Effective: Systolic blood pressure decreased < 30 mmHg and diastolic blood pressure decreased < 10 mmHg, the clinical symptoms of HTN were relieved, and the dosage of antihypertensive drugs was reduced. ④. Invalidity: The clinical symptoms of HTN were not relieved, and the systolic and diastolic blood pressures were not changed or even increased compared with those before operation.

Effectiveness = (cure+ marked effect+ effective)/total number× 100%.

### Statistical methods

Data was analyzed using statistical product and service solutions software version 22.0. Continuous variables were expressed as mean ± standard deviation or median. Differences between variables were evaluated using the independent samples t-test. Categorical variables were expressed as a ratio (%), and differences between variables were compared using the chi-squared test. Data was deemed significant if *P* < 0.05 for all tests.

## Results

1. Comparison of basic data of two groups: There was no significant difference in the age, sex, course of HTN, duration of TN, grade of HTN, preoperative systolic and diastolic blood pressure between the experimental group and the control group (*P* > 0.05), as shown in Table 1.
2. The symptoms of TN disappeared after operation in all patients.

**Table 1:** Comparison of clinical data between the two groups

	Control Group (*n* = 28)	Experimental Group(*n* = 30)	*P* value
Gender			0.266
Male	18	17	
Female	10	13	
Age (year)	61.2 ± 9.6	60.9 ± 9.4	0.582
Course of TN (year)	11.5 ± 3.3	11.3 ± 3.1	0.749
Course of HTN (year)	20.5 ± 10.5	19.8 ± 11.2	0.807
Classification of HTN			0.837^a^
Level I	3	4	
Level II	10	9	
Level III	15	17	
Preoperative BP			
SBP (mmHg)	181.0 ± 20.4	181.3 ± 20.3	0.815
DBP (mmHg)	108.9 ± 8.9	108.2 ± 10.2	0.756

^a^From the merger of level II and III

**Fig. 1:**
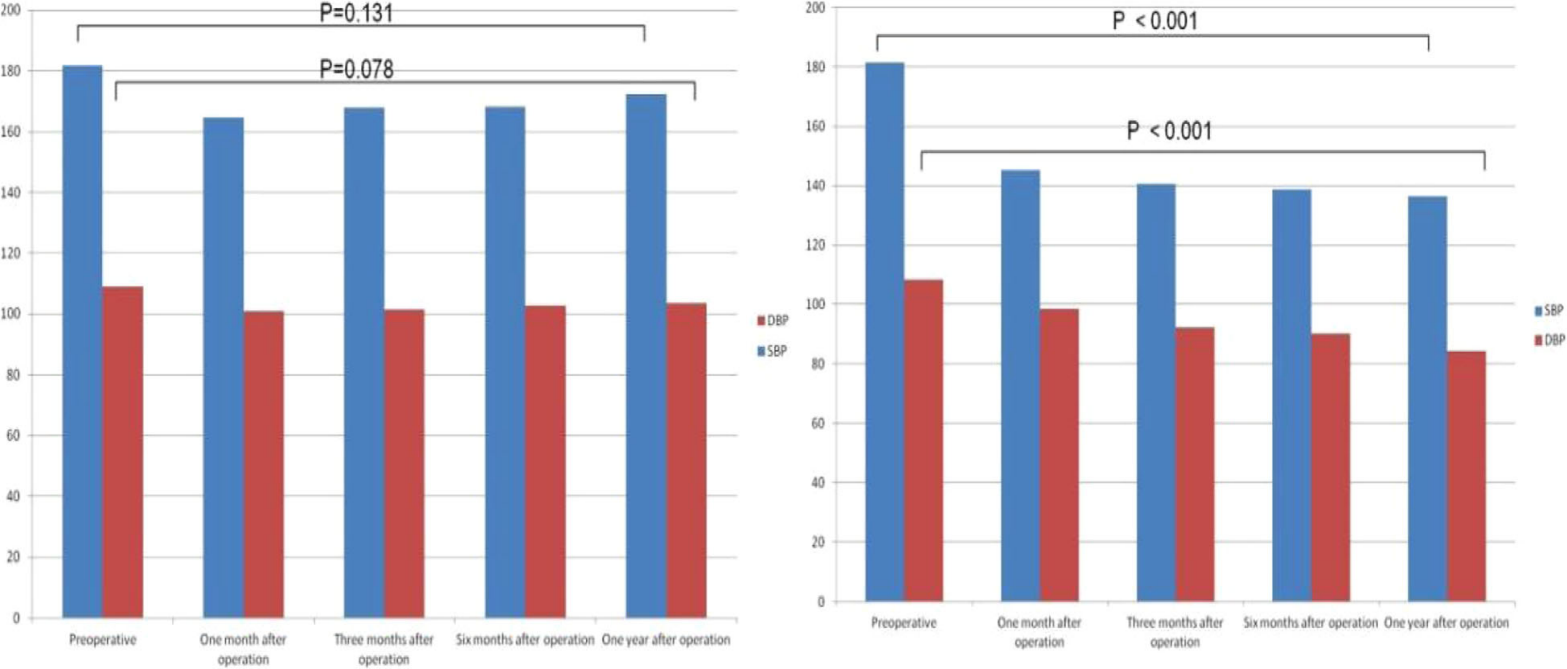
Changes of BP before and after operation in two groups (mmHg)

The changes of blood pressure after operation are shown in Table 2 and Fig. 1. The blood pressure of the two groups was lower than that before operation. There were no significant differences in SBP and DBP between the preoperative and the 12 months after surgery in the control group. SBP and DBP in the experimental group decreased significantly at 12 months after operation compared with those before operation, and the difference was statistically significant.

3. According to the blood pressure after operation, the control group: 2 cases (7.1%) were cured, 3 cases (10.7%) were marked effect, 4 cases (14.3%) were effective, 19 cases (67.9%) were invalidity, and the effective rate was 32.1%. In the experimental group, 10 cases (33.3%) were cured, 9 cases (30%) were marked effect, 6 cases (20%) were effective and 5 cases (16.7%) were invalidity. The effective rate was 83.3%, as shown in Table 3.

**Table 2:** Changes of BP before and after operation in two groups (mmHg)

Time	Control Group		Experimental Group	
	SBP	DBP	SBP	DBP
Preoperative	181.0 ± 20.4	108.9 ± 8.9	181.3 ± 20.3	108.2 ± 10.2
One month after operation	164.3 ± 17.8	100.8 ± 11.2	145.3 ± 10.8	98.4 ± 12.5
Three months after operation	167.8 ± 17.9	101.5 ± 13.4	140.6 ± 14.2	92.1 ± 10.7
Six months after operation	168.2 ± 18.3	102.7 ± 15.8	138.8 ± 12.1	90.2 ± 11.3
One year after operation	172.4 ± 21.6	103.6 ± 12.8	136.5 ± 11.6	84.5 ± 7.2
△BP	8.6 ± 5.8	5.3 ± 7.7	44.8 ± 9.5	23.7 ± 9.7
P value	0.131	0.078	<0.001	<0.001

△BP:Changes of blood pressure before and after operation

**Table 3:** Comparisons of surgical effects between two groups

Curative Effect	Control Group(%)	Experimental Group(%)
Cure	2(7.1)	10(33.3)
Marked Effect	3(10.7)	9(30)
Effective	4(14.3)	6(20.0)
Invalidity	19(67.9)	5(16.7)

## Discussion

HTN is one of the most common chronic diseases that threaten human health. It is also a major risk factor for cardiovascular and cerebrovascular diseases. Its complications, such as cerebral hemorrhage, have high disability and mortality rates. Therefore, the prevention and control of HTN is of great significance to the prevention of cardiovascular and cerebrovascular diseases. At present, the treatment of HTN is mainly drug treatment. However, some of the hypertensive patients only use antihypertensive drugs, the effect is often unsatisfactory. In 1979, Jannetta et al. [2] found that part of HTN was caused by abnormal arterial compression of the left RVLM and REZ area, which was called neurogenic HTN. After MVD in these hypertensive patients, blood pressure returned to normal in some patients, and some patients’ blood pressure decreased significantly. It provides a new treatment for HTN. Since then, a large number of clinical studies have proved that MVD can significantly reduce blood pressure in patients with neurogenic HTN, or even reduce the types and dosage of antihypertensive drugs, or discontinue antihypertensive drugs [5, 6].

At present, although MVD treatment of neurogenic HTN has been recognized by international scholars, but there are few patients with neurogenic HTN treated with MVD. The main reason is that there is no unified criterion for screening and diagnosis of neurogenic HTN patients. More importantly, most patients are unwilling to accept craniotomy because of HTN. However, MVD has been widely recognized in the treatment of TN. At the same time, through clinical observation, TN can also cause and/or aggravate HTN, which is mainly caused by long-term repeated pain stimulation, mental tension, anxiety, which makes the sympathetic nerve excited. Sympathetic nerve excitation not only acts on myocardial B receptor to accelerate heart rate and strengthen myocardial contraction, but also acts on peripheral blood vessel a receptor to make small arteries contract and peripheral blood vessel resistance increase, leading to increased blood pressure. In addition, long-term sympathetic nerve excitation can release norepinephrine through hypothalamus and peripheral sympathetic nerve endings, which can increase blood pressure [7] After MVD, the symptoms of TN disappeared, and then the tension and excitement smoothed, the sympathetic excitability gradually decreased to normal, and the blood pressure also decreased to normal [8]. Therefore, although there are abnormal blood pressure compressions in RVLM and REZ regions, repeated and long-term pain stimulation is also one of the causes of neurogenic HTN [7]. We found that patients with TN with HTN only performed trigeminal MVD, and their blood pressure decreased in varying degrees. The cure rate was 2 cases (7.1%) and marked effective was 3 cases (10.7%) and 4 cases (14.3%) were effective and 19 cases (67.9%) were invalidity. The total effective rate was 32.1%.

Because the left medulla oblongata is oppressed by arteries and long-term pain stimulation in patients, which can lead to over excitation of sympathetic nervous system and hypertension [9]. Therefore, we believe that patients with neurogenic HTN combined with TN is the best surgical indications. For patients with trigeminal neuralgia combined with hypertension, the left trigeminal nerve and RVLM and REZ regions can be performed simultaneously, and the patients can obtain ideal curative effect and benefit. At present, the curative effect of MVD in the treatment of neurogenic HTN is different. The highest effective rate is 87.5%, and the lowest is 23.8% [10]. Sindou et al. [11] conducted a retrospective study of 48 patients underwent MVD, 14 patients recovered normal blood pressure after operation, 14 patients were marked effect, 10 patients were effective. The total effective rate was 79.2%. Geiger [12] et al. performed MVD in patients with neurogenic HTN, the postoperative effective rate was 87.5%. We performed MVD on the trigeminal nerve and the left RVLM and REZ area simultaneously, and the blood pressure of the patients decreased effectively, the cure was: 10 cases (33.3%), markedly effect: 9 cases (30%), effective: 6 cases (20%), invalidity: 5 cases (16.7%). The total effective rate was 83.3%. Compared with preoperative blood pressure, the change of blood pressure has significant statistical difference. The symptoms of cranial nerve compression disappeared and no serious complications occurred in all patients, which further confirmed the efficacy of MVD in the treatment of neurogenic HTN. And our efficiency is consistent with international research results.

Because most of the patients with primary TN complicated with neurogenic HTN are the elderly, they are more concerned about the risk of surgery. If the relationship between trigeminal nerve and medulla oblongata and peripheral blood vessels can be fully evaluated before operation, and the responsible blood vessels can be initially determined, it will be conducive to the operation. In 3D-TOFMRA sequence images, the nerve and brain tissue showed equal signals, the peripheral blood vessels showed high signals, and the cerebrospinal fluid showed low signals. The contrast was clear. The relationship between peripheral blood vessels and nerves and brainstem could also be observed directly. The maximum density projection technique could also be used to realize arterial imaging to understand the origin and course of the responsible blood vessels [13]. It can provide an effective means for preoperative evaluation and screening.

A large number of clinical studies have confirmed that patients with neurogenic HTN are due to the compression of the left RVLM and REZ neurovascular areas [14–16]. MVD to relieve the vascular compression in the left RVLM and REZ is the treatment of etiology. Therefore, the patients we selected were all left TN patients with left RVLM and REZ neurovascular compression. Whether the right HTN RVLM and REZ neurovascular compression patients with MVD have the same efficacy requires further clinical study. Similarly, the long-term follow-up evaluation of MVD for hypertensive patients needs to be determined by a rigorous, multi-center, prospective and randomized clinical study. Only in these areas has made great progress, microvascular decompression can play an important role in the treatment of neurogenic HTN.

## Conclusions

MVD is an effective treatment for neurogenic HTN. However, the criteria for selecting hypertensive patients still need to be further determined. In order to improve the efficacy of neurogenic HTN surgery, including the following indications as far as possible: left TN; neurogenic HTN; abnormal blood pressure compression in the left RVLM and REZ areas in high-resolution MR images; the blood pressure in these patients cannot be effectively controlled by combined antihypertensive drugs.

## Data Availability

The datasets used or analysed during the current study are available from the corresponding author on reasonable request.

## Abbreviations

3D-TOFMRA: three-dimensional time-of-flight magnetic resonance angiography
BP: Blood pressure
DBP: Diastolic blood pressure
HTN: Hypertension
MRI: Magnetic resonance imaging
MVD: Microvascular decompression
REZ: The posterior cranial nerve outgoing (entering) brainstem
RVLM: Rostral ventrolateral medulla
SBP: Systolic blood pressure
TN: Trigeminal neuralgia
